# Biological and toxicological evaluation of *Rhus trilobata* Nutt. (*Anacardiaceae*) used traditionally in mexico against cancer

**DOI:** 10.1186/s12906-019-2566-9

**Published:** 2019-07-01

**Authors:** Luis Varela-Rodríguez, Blanca Sánchez-Ramírez, Ivette Stephanie Rodríguez-Reyna, José Juan Ordaz-Ortiz, David Chávez-Flores, Erika Salas-Muñoz, Juan Carlos Osorio-Trujillo, Ernesto Ramos-Martínez, Patricia Talamás-Rohana

**Affiliations:** 1 Departamento de Infectómica y Patogénesis Molecular, CINVESTAV-IPN, Ave. Instituto Politécnico Nacional No. 2508, Col. San Pedro Zacatenco, C.P. 07360 Ciudad de México, Mexico; 2 Facultad de Ciencias Químicas, Universidad Autónoma de Chihuahua, Circuito No. 1, Nuevo Campus Universitario, C.P. 31125 Chihuahua, Chih. Mexico; 3 Laboratorio de Metabolómica y Espectrometría de Masas, Unidad de Genómica Avanzada, CINVESTAV-IPN, Libramiento Norte Carretera Irapuato-León Km. 9.6, C.P. 36824 Irapuato, Gto. Mexico; 4 Departamento de Anatomía Patológica, Hospital CIMA, Av. Hacienda del Valle No. 7120, Fraccionamiento Plaza las Haciendas, C.P. 31217 Chihuahua, Chih. Mexico

**Keywords:** Acute toxicity, Biological activity, *β*-PGG, Colorectal adenocarcinoma, Phytochemical composition, *Rhus trilobata*

## Abstract

**Background:**

*Rhus trilobata* Nutt. (*Anacardiaceae*) (RHTR) is a plant of Mexico that is traditionally used as an alternative treatment for several types of cancer. However, the phytochemical composition and potential toxicity of this plant have not been evaluated to support its therapeutic use. Therefore, this study aimed to evaluate the biological activity of RHTR against colorectal adenocarcinoma cells, determine its possible acute toxicity, and analyze its phytochemical composition.

**Methods:**

The traditional preparation was performed by decoction of stems in distilled water (aqueous extract, AE), and flavonoids were concentrated with C_18_-cartridges and ethyl acetate (flavonoid fraction, FF). The biological activity was evaluated by MTT viability curves and the TUNEL assay in colorectal adenocarcinoma (CACO-2), ovarian epithelium (CHO-K1) and lung/bronchus epithelium (BEAS-2B) cells. The toxicological effect was determined in female *BALB/c* mice after 24 h and 14 days of intraperitoneal administration of 200 mg/kg AE and FF, respectively. Later, the animals were sacrificed for histopathological observation of organs and sera obtained by retro-orbital bleeding for biochemical marker analysis. Finally, the phytochemical characterization of AE and FF was conducted by UPLC-MS^E^.

**Results:**

In the MTT assays, AE and FF at 5 and 18 μg/mL decreased the viability of CACO-2 cells compared with cells treated with vehicle or normal cells (*p* ≤ 0.05, ANOVA), with changes in cell morphology and the induction of apoptosis. Anatomical and histological analysis of organs did not reveal important pathological lesions at the time of assessment. Additionally, biochemical markers remained normal and showed no differences from those of the control group after 24 h and 14 days of treatment (*p* ≤ 0.05, ANOVA). Finally, UPLC-MS^E^ analysis revealed 173 compounds in AE-RHTR, primarily flavonoids, fatty acids and phenolic acids. The most abundant compounds in AE and FF were quercetin and myricetin derivates (glycosides), methyl gallate, epigallocatechin-3-cinnamate, *β*-PGG, fisetin and margaric acid, which might be related to the anticancer properties of RHTR.

**Conclusion:**

RHTR exhibits biological activity against cancer cells and does not present adverse toxicological effects during its in vivo administration, supporting its traditional use.

**Electronic supplementary material:**

The online version of this article (10.1186/s12906-019-2566-9) contains supplementary material, which is available to authorized users.

## Background

Plants have traditionally been used in alternative medicine for the prevention and treatment of diseases that afflict the general population because of their active compounds (ACs) [1, 2]. However, their importance is not only based on their pharmacological or chemotherapeutic effects but also on the possibility they offer to develop new drugs based on the structures of their components [3]. In fact, a significant number of commercial drugs have been developed directly from plants, such as quinine from *Cinchona officinalis* for the treatment of malaria [4], morphine from *Papaver somniferum* as an analgesic [5], and Taxol from *Taxus brevifolia* as an anticancer agent [6], among others. Therefore, countries with a great biodiversity of plants have become suitable places for the identification of new ACs with medicinal properties. Currently, Mexico ranks fifth in plant diversity and uses approximately 7000 species for medicinal purposes, although pharmacological validation has only been carried out for 5% of them [7]. Consequently, research designed to determine the efficacy of many plants that are used as alternative medicine and to evaluate their potential as a source of new ACs with therapeutic activity is important [8]. *Rhus trilobata* Nutt. (RHTR) is a plant of the state of Chihuahua in Mexico, which has been used for the treatment of gastrointestinal diseases and cancer. Scientific information about RHTR medicinal use and possible pharmacological properties is scarce. Currently, phytochemical constituents of the sumac RHTR have not been fully investigated. Abbott et al., (1966) showed the antineoplastic activity of extracts elaborated by the maceration of leaves with EtOH:CHCl3 (1:1) (100 mg/kg) and by the decoction of fruits in water (400 mg/kg), when administered intraperitoneally (i.p.) as a fixed dose for 7 days to Syrian hamsters xenotransplanted with human duodenum adenocarcinoma cells. In both cases, a 33% reduction in the weight of the tumors was observed with respect to the control condition without treatment [9].

Later, Pettit et al. (1974) were able to isolate gallic acid and ethyl gallate from a 50% ethanolic extract of RHTR leaves by column chromatography. Then, using the human nasopharyngeal adenocarcinoma cell line KB, they evaluated the biological activities of these extracts in vitro*,* which presented an IC_50_ of 3.1 and 18 μg/mL, respectively, from which they assigned cytotoxic activity of the plant to both compounds [10]. The results obtained by different research groups have demonstrated the presence of compounds with antineoplastic activity in other species of the *Rhus* genus, such as protocatechuic acid, fustin, fisetin, sulfuretin, and butein in *R. verniciflua* [11] and heptadecatrienylhydroquinone derivates in *R. succedanea* [12]. In addition, other polyphenols with antioxidant activity have been identified, such as garbanzol, quercetin, gallic acid and amentoflavone, which have been shown to have wide applications in health by combating processes associated with oxidative stress, such as cardiovascular and neurodegenerative diseases and cancer [13]. Therefore, it is possible that the cytotoxic activity observed in RHTR is due to the presence of other compounds that are distinct from gallates. Despite these beneficial compounds, some studies performed with *R. toxicodendron*, *R. radicans,* and *R. diversiloba* have demonstrated that the resin secreted by these plants can generate contact dermatitis when applied to the skin due to the presence of the urushiol compound, which can cause death in people who are sensitive to its effects [14–16]. Despite a dearth of studies in which animal models have been used to analyze the antineoplastic activity of RHTR [9, 10], the toxic and/or biochemical effects of the extracts in animal models have not been reported. Thus, additional toxicological studies are required to examine the different *Rhus* species to validate their therapeutic use.

Therefore, the aim of this study was to determine the biological activity of RHTR in colorectal adenocarcinoma cells to demonstrate the antineoplastic activity of the aqueous extract of the plant or its flavonoid fraction, as well as to evaluate the toxicological effect of RHTR in a murine model and, finally, the phytochemical composition of RHTR to corroborate its medicinal properties.

## Methods

### Plant material

**Fig. 1:**
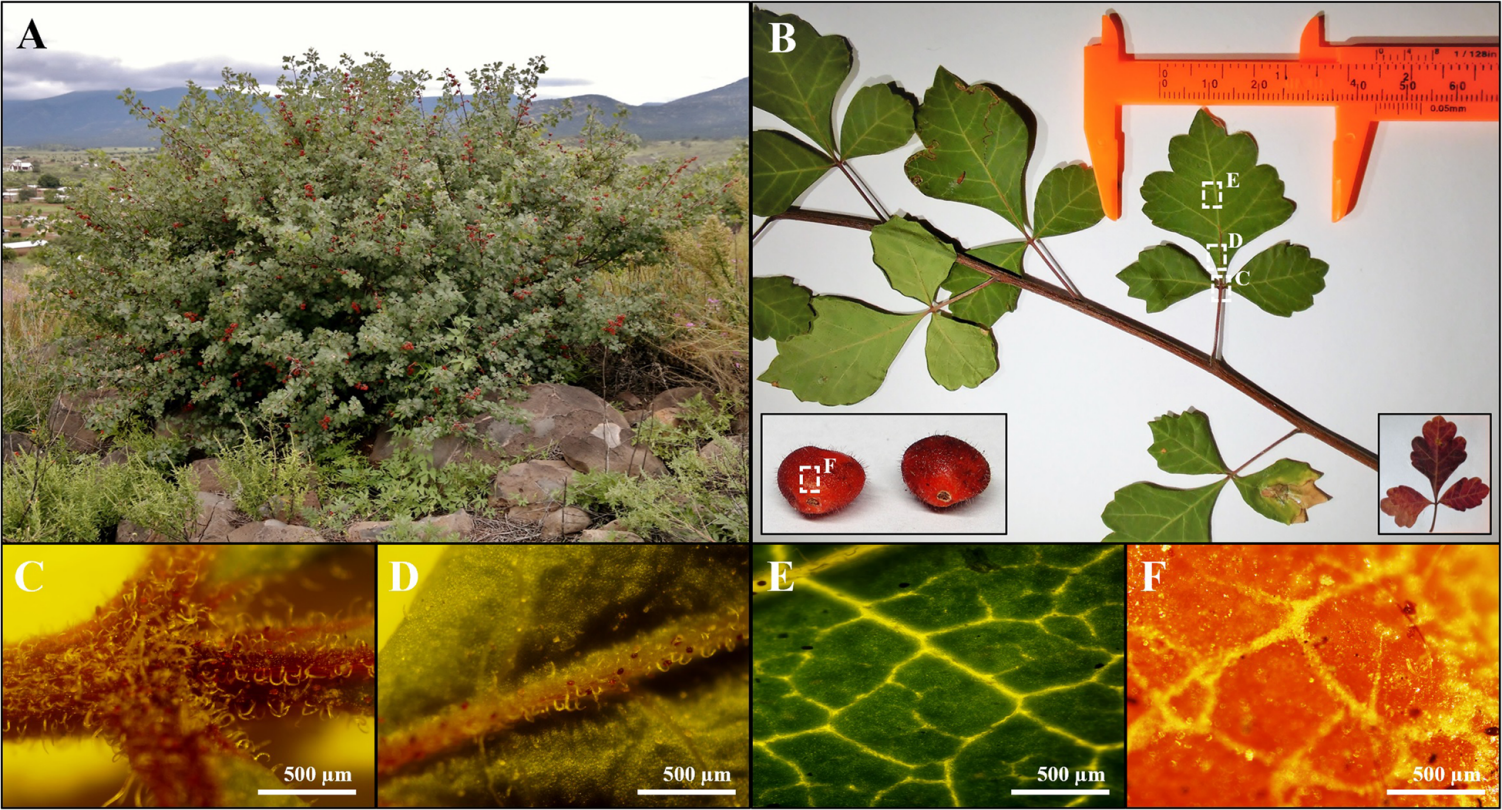
Morphological characteristics of RHTR. Representative characteristics of specimens collected from the Cerro Pelón in Namiquipa Township (Chihuahua, México) in May 2014 (**a**). Trifoliate leaves, pinnately composed, serrated edges and evidently split, with a red-orange color in the autumn season were observed, and equally, small fruits, spherical, fleshy and reddish were found under a stereoscope at 2X magnification (**b**). **c**–**f** Areas analyzed by microscopy at 4X magnification: adaxial face of leaves with axillary buds covered with simple trichomes (**c**-**e**) and epicarp of an irregular fruit covered with microvilli and resin (**f**). (Photographs are the property of Luis Varela-Rodríguez and Blanca Sánchez-Ramírez, all copyright reserved)

The stems of *Rhus trilobata* Nutt. (*Anacardiaceae*) were collected from Cerro Pelón, Municipality of Namiquipa (Chihuahua, Mexico) (INEGI topographic map H13C42 and geographical GPS coordinates: 29°5′59″N, 107°32′33″W to 1960 m.a.s.l.), in May 2014, at the beginning of the spring season according to the popular use of the plant; no complete specimens and/or roots were collected to allow regeneration. The collected material was transported to the laboratory protected from light; leaves and fruits were removed. Stems were washed with tap water, dried for 48 h at room temperature, and milled to a sieving size of 0.5 mm; finally, the stems were lyophilized. Features found in RHTR that allowed its identification were the presence of trifoliate leaves, pinnately composed, serrated edges and evidently cleaved, as well as the presence of small, spherical, reddish, fleshy fruits with a characteristic sour taste (Fig. 1) [17, 18]. The above features correspond to those described in the herbarium G.B. Hinton (No. 18713), found online at the *Irekani*, property of Unidad de Informática para la Biodiversidad (UNIBIO) from Instituto de Biología-UNAM [19], as well as in the virtual herbarium CONABIO (No. K000081429) where similarities in the shape and arrangements of the leaves and fruits can be seen [20]. The identification of the collected specimens was performed by Toutcha Lebgue Keleng, a Ph.D. of the Facultad de Zootecnia y Ecología from UACH. The identified specimen was validated by Dr. María de la Luz Arreguín Sánchez, herbarium curator of the Escuela Nacional de Ciencias Biológicas from IPN, and by Biol. Laura de Léon Pesqueira of the Instituto de Ciencias Biomédicas from UACJ (Voucher No. 1543).

### Preparation of plant extracts and fractionation

The traditional preparation of RHTR was performed by decoction with 25 g of stems in 500 mL of boiling distilled water for 30 min (aqueous extract; AE). Subsequently, the AE was filtered and centrifuged at 2500 rpm for 15 min at 4 °C in conical tubes to recover the supernatants and concentrated under negative pressure with a rotary vacuum evaporator at 40 °C and 5 rpm (Rotavapor® R-300, Büchi). Next, the AE was freeze-dried (FreeZone Triad, Labconco®) and stored at − 20 °C in an amber vial. The fractionation of AE was performed by solid phase extraction with the aid of a vacuum Manifold (Visiprep™, Sigma®) and ENVI™-C_18_ cartridges (Supelclean™, Sigma®) to concentrate compounds of a flavonoid nature. The cartridge was activated with 20 mL of MeOH (J.T. Baker®) and 1% acetic acid in water. Subsequently, 15 mL of ethyl acetate (flavonoid fraction, FF) HPLC-grade from J.T. Baker® was eluted through the cartridge and collected for concentration by rotary evaporation. Finally, the fraction was weighed and resuspended in 2 mL of 50% MeOH (MS grade, J.T. Baker®) and filtered with a 0.20 μm PTFE syringe filter (Corning®) for storage as a stock solution in an amber vial at − 20 °C for later use. Extracts and fractions were prepared according to the method disclosed in the Mexican patent: MX/E/2018/078316.

#### Physicochemical analysis

The determination of the parameters established in the quality control methodology for plant material by WHO was carried out with RHTR samples [21], as well as the quantification of primary metabolites by spectrophotometry with phenol-sulfuric acid (carbohydrates) [22], Bradford (proteins) [23] and Ruhemann (amino acids) [24] assays, respectively.

#### Quantification of polyphenols and antioxidants

Total phenolic content and elimination activity of free radicals in RHTR was estimated by Folin-Ciocâlteu (Sigma®) and 2,2-diphenyl-1-picrylhydrazyl (DPPH, Sigma®) assays [25, 26]. Additionally, flavonoids and anthocyanins were quantified using aluminum chloride (Sigma®) and differential pH methods, respectively [27, 28]. Gallic acid (Ga, Sigma®), 6-hydroxy-2,5,7,8-tetramethylchroman-2-carboxylic acid (Trolox, Sigma®), quercetin (Que, Sigma®) or cyanidin-3-glucoside (Cya) was used as a standard compound to calculate the concentration as an equivalent. Antioxidant activity was considered high when the antioxidant efficient index (AEI) was ≥1. The half antioxidant concentration (AC_50_) and AEI were calculated according to the following formulas [29]: *AC*_*50*_ *= [(absorbance control – absorbance sample) / (absorbance control)] × 100*, and *AEI = 1 / (AC*_*50*_*) x (Time AC*_*50*_*),* respectively*.*

### Cell cultures

The cell lines used for this study were colorectal adenocarcinoma cells CACO-2 (HTB-37™), Chinese hamster ovary cells CHO-K1 (CCL-61™) and lung/bronchus human epithelial BEAS-2B cells (CRL-9609™) acquired from ATCC®. Cell monolayers were maintained in McCoy’s 5A (Gibco™) and Dulbecco’s Modified Eagle’s Medium (DMEM, Gibco™) supplemented with 10% (*v/v*) fetal bovine serum (FBS, Gibco™) thermally decomplemented, and 1% penicillin–streptomycin (10 mg/mL, Sigma®) and gentamycin (10 μg/mL, Sigma®). The cells were incubated at 37 °C with 5% CO_2_ (95% humidity) and harvested using 1X trypsin-EDTA solution (Sigma®); cell viability was determined using the Trypan blue (0.4%, Sigma®) exclusion assay.

### Biological activity of RHTR in colon cancer and normal cells

RHTR activity in cell lines was analyzed using dose-response viability curves with 3-(4,5-dimethylthiazol-2-yl)-2,5-diphenyltetrazolium bromide (MTT, Sigma®) [30]. For this purpose, 1 × 10^4^ cells were seeded in 96-well plates (Corning®) in supplemented medium and incubated for 24 h, at which time treatments with RHTR extract and fraction (concentrations from 5 to 1000 μg/mL) were added; cultures treated with vincristine, an anti-cancer drug (Sigma®) or 1X PBS were used as positive or negative controls, respectively. Cultures were incubated for 24 h, and 4 h prior to the end of the treatments, 20 μL of MTT (5 mg/mL in 1X PBS, Sigma®) was added to each well. At the end of the experiment, cell lysis was conducted with acidified isopropanol (Sigma®), and the absorbance at 590 nm was measured using a Varioskan® Flash microreader (Thermo Scientific®). The cell viability was calculated using the following formula: *% Viability = (absorbance sample / absorbance control) × 100.* The half-maximal inhibitory concentration (IC_50_) was calculated by regression analysis (percent survival vs log concentration). The biological activity in cancer cells was classified as follows [31]: high IC_50_ ≤ 30 μg/mL, medium 31–70 μg/mL and low 71–100 μg/mL. Finally, for the antiproliferative assays, CACO-2 cells were treated every 24 h with the IC_50_ previously determined up to 72 h to determine the mitotic index (MI).

#### Selectivity index (SI) and median lethal dose (LD_50_)

The degree of selectivity [32] and lethal dose [33] were determined based on the IC_50_ obtained from in vitro anticancer and toxicological assays. The formulas applied were as follows: *SI = IC*_*50*_ *normal cell line / IC*_*50*_ *cancer cell line* and *LD*_*50*_ *(oral, mg/kg) = 10*^*EXP*^ *[(0.372 x log IC*_*50*_ *normal cell line) + 2.024].*

#### Measurement of the cell cycle

The cell cycle and sub-G_1_ distribution were determined by flow cytometry with propidium iodide staining in CACO-2 cells treated with RHTR [2, 34]. To achieve this goal, 5 × 10^5^ cells per well were seeded in a 6-well plate (Corning®) with supplemented medium for 24 h. The adherent cells were treated with the IC_50_ of samples and controls (20 μg/mL mitomycin Sigma® and 1X PBS vehicle) for 24 h. After this period, the culture medium was removed, and the cells were detached by trypsinization. Subsequently, the cells were fixed and permeabilized with 50% EtOH at − 20 °C for 12 h. Finally, the cells were washed and pelleted by centrifugation (2000 rpm, 10 min and 4 °C), followed by the addition of 400 μL 1X PBS, 10 μL RNAse A (10 mg/mL, Sigma®) and 20 μL propidium iodide (1 mg/mL, Invitrogen®) for 1 h at 37 °C in the dark. The relative DNA content in the cells was analyzed with a BD FACSCalibur™ (Becton Dickinson®) based on red fluorescence, and quantitation of each cell cycle stage was performed with ModFit LT software (version 5.0, Verity Software House®).

#### Genotoxic analysis by the comet assay

The genotoxic activity of RHTR was evaluated with a single cell gel electrophoresis method under alkaline conditions using the CHO-K1 cell line [35, 36]. To achieve this goal, 5 × 10^4^ cells per well were seeded in a 24-well plate (Corning®) with 2 mL of supplemented McCoy’s 5A medium for 24 h. Adherent cells were treated with the IC_50_ of samples and controls (0.3% H_2_O_2_ Sigma® and 1X PBS vehicle) for 24 h, the culture medium was removed, the cell monolayer was washed with 1X PBS and the cells were detached by trypsinization. After centrifugation, the cells were mixed with 1% low melting point agarose (Sigma®) and rapidly spread onto a microscope slide that had been precoated with 0.5% normal melting point agarose (Sigma®). To stain nucleic acids, SYTOX® green (5 mM, Thermo Scientific®) at a 1:5000 dilution (*v/v*) was added to the buffers. The slides were coverslipped and allowed to jellify. Later, the coverslips were placed in ice-cold lysis buffer (2.5 M NaCl, 100 mM EDTA, 10 mM Tris-base, 10% DMSO and 1% Triton X-100 of Sigma®, pH 10) for 1 h at 4 °C. After lysis, the slides were placed in a horizontal electrophoresis chamber with alkaline buffer (1 mM EDTA and 300 mM NaOH of Sigma®, pH 13) for 30 min at 4 °C in the dark to allow DNA unwinding. Electrophoresis was carried out for 20 min at 25 V and 300 mA, and the slides were then immersed in neutralization buffer (0.4 M Tris-HCl Sigma®, pH 7.5) for 15 min at 25 °C and fixed with 2 mL absolute EtOH (J.T.Baker®) for 10 min in the dark. Finally, the preparations were mounted with Vectashield® medium (Vector Laboratories®), and the length of damaged DNA migration was analyzed and measured by confocal microscopy (LSM 700, Zeiss®) using ZEN 2011 software (version 1.0, Zeiss®). The genotoxic activity was classified as high if 40–95% of the cells showed damaged DNA, medium if 10–40% of them had damaged DNA, low when 5–10%, and null when less than 5% had damaged DNA.

#### ROS-intracellular quantification by CM-H_2_DCFDA

The production of intracellular reactive oxygen species (ROS) was determined with the chemical reporter (5-(and-6)-chloromethyl-2′,7′-dichlorodihydrofluorescein diacetate (CM-H_2_DCFDA) in CHO-K1 cells treated with RHTR [37]. To achieve this goal, 1 × 10^4^ cells per well were seeded in 96-well plates (Corning®) with 200 μL of supplemented McCoy’s 5A medium for 24 h. The adherent cells were treated with the IC_50_ of samples and controls (0.3% H_2_O_2_, Sigma® and 1X PBS vehicle) for 24 h. After this period, the culture medium was removed, and 25 μM CM-H_2_DCFDA (Thermo Scientific®) was added for 30 min at 37 °C. Finally, the fluorescence was quantified at λ_ex_ 495 (9 bandwidth) / λ_em_ 530 (20 bandwidth) nm in a Varioskan® Flash microreader (Thermo Scientific®) and corroborated by confocal microscopy (LSM 700, Zeiss®).

#### Evaluation of morphology and cell death

Adherent cultures plated at 3 × 10^4^ cells per well in Lab-Tek™ chamber slides (Thermo Scientific®) were treated with the IC_50_ of samples and controls (25 μg/mL vincristine Sigma® and 1X PBS vehicle) for 24 h. Then, the culture medium was removed, and the cells were fixed with 4% paraformaldehyde (Sigma®) for 1 h at 37 °C. The cells were then washed with 1X PBS and stained. The morphology of the cells was determined using Hemacolor® rapid staining (Merck®), and the presence of apoptosis was assessed using the Apo-BrdU™ TUNEL assay kit with Alexa Fluor 488 (Invitrogen®), according to the manufacturer’s instructions. After applying both stains, the slides were treated with Vectashield®/DAPI mounting medium (Vector Laboratories®) or Entellan® resin (Merck®) according to the assay and analyzed by optical microscopy (BX41, Olympus®) or confocal microscopy (LSM 700, Zeiss®) using a 40X immersion lens with Image-Pro Plus (version 4.0, Media Cybernetics©) or ZEN 2011 (version 1.0, Zeiss®) software.

### Experimental animals

According to the recommendations of OECD [38], 30 adult female *BALB/c* mice with a body weight of 25 ± 5 g and aged 6 to 8 weeks were obtained from the Bioterium of the Facultad de Ciencias Químicas, UACH (Additional file 1: Figure S1A). This study was carried out in accordance with Official Mexican Regulations [39] and approved by an Ethics Committee (Authorization No. 0010/11).

### Acute toxicity studies

The toxicological effects of RHTR were determined in the mouse animal model using Guideline 425 by OECD with some modifications [40]. For this purpose, female *BALB/c* mice were randomly divided into two studies with three groups each (5 animals per group) (Additional file 1: Figure S1B). Subsequently, a single dose of 200 mg/kg body weight of sample and 100 μL of vehicle (1X PBS) was administered by the intraperitoneal route (i.p.) (Additional file 1: Figure S1C); three groups of animals were sacrificed after 24 h (Study 1), and the other three groups were sacrificed after 14 days (Study 2, Additional file 1: Figure S1). Animals were observed at 1, 2, 4 and 6 h posttreatment to detect any signs of toxicity and/or death at 24 h (Additional file 1: Figure S1D). Mice were fasted for 8 h before finishing the assay to avoid biochemical alterations due to food, but access to water was maintained ad libitum (Additional file 1: Figure S1E). The LD_50_ < 200 mg/kg was considered when 3 individuals died in a group and the LD_50_ > 200 mg/kg when one or none of the individuals died (Additional file 1: Figure S1F). The weight of the mice was determined using an electronic bascule (CS200, Ohaus®) at the beginning and end of the treatments. The % body weight change (% BWC) and body mass index (BMI) were calculated as follows [41]: *% BWC = [(final weight – initial weight) / initial weight] × 100* and *BMI = weight (g) / length of nose until rear foot (cm)*^*2*^*.* After the evaluation time, the surviving animals were anesthetized by i.p. injection with 250 μL of diluted sodium pentobarbital 1:10 (*v/v*) (6.3 g/100 mL, Pet’s Pharma®) using an insulin syringe and 25 G needle for the subsequent analysis (Additional file 1: Figure S1G).

#### Determination of biochemical parameters and hematic biometry

Peripheral blood samples were obtained by retro-orbital bleeding of anaesthetized animals using capillary tubes and BD Microtainer® tubes with K_2_EDTA (for cell counts) without anticoagulant (to obtain serum for biochemical determinations) (Additional file 1: Figure S1H). Hematic biometry was performed using the hematology autoanalyzer system (BC-2300, Mindray®) and the biochemical parameters with the standard diagnostic test (Human® kits) with an automated medical system (Prestige® 24i, Tokyo Boeki®).

#### Anatomical and histological assessments

After blood samples were obtained, mice were sacrificed by cervical fracture and mounted on a dissecting board for necropsy. A complete mid-laparotomy was performed, and lungs, spleen, heart, liver and kidneys were dissected for anatomical observations (Additional file 1: Figure S1I). Adhesions and/or connective tissue in organs were eliminated; organs were measured using a digital Vernier caliper (Truper®), weighed, and photographed. The relative organ weight expressed as percentage (% ROW) was calculated as follows: *% ROW = [(organ weight / body weight mouse) × 100].* Finally, organs were fixed in 4% paraformaldehyde (Sigma®) and embedded in paraffin to obtain 5-μm-thick sections. Tissue slices were stained with hematoxylin-eosin (Merck®) to perform the microscopic analysis searching for pathological lesions by optical microscopy (BX41, Olympus®) with 10X and 40X objectives.

### Phytochemical characterization of RHTR by UPLC-MS^E^

The identification of compounds in RHTR sumac was performed by UPLC (Acquity™ series, Waters®) with a photodiode array detector (PAD) and coupled to MS (Synapt™ G1, Waters®). The instrument was equipped with an Acquity™ UPLC CSH C_18_ column (2.1 mm × 150 mm, 1.7 μm, Waters®); the mobile phase used was Milli-Q purified water (solvent A) (Simplicity® UV, Millipore®) and acetonitrile (solvent B) (J.T.Baker®) (both acidified with 0.1% formic acid, *v/v*) MS-grade. The solvents were degassed by sonication in an ultrasonic bath (Branson 1800, Emerson™). The compounds were eluted via a gradient separation in reversed-phase as follows: 0 min, 5% B; 0.5 min, 5% B; 20 min, 75% B; 25 min, 75% B; 25.5 min, 90% B and holding for 2 minutes for column washing; 27.6 min, 5% B and holding for 4.4 min for column re-equilibration. Samples (1 mg/mL) were filtered with a 0.20-μm PTFE syringe filter (Captiva Econo Filter, Agilent®) and maintained at 4 °C during the assay. The chromatographic conditions were as follows: the flow rate was set at 0.2 mL/min throughout the gradient from the UPLC system into the MS detector; the injection volume was 10 μL; and the column temperature was maintained at 30 °C. The samples were analyzed by PAD at 280 nm and ions generated by the electrospray ionization source (ESI) in negative and positive mode. Spectra were acquired over a mass range from 50 to 1500 m/*z* using the MS^E^ acquisition mode. The precursor ion collision energy was set to 6 eV (trap section) and 20 to 40 eV in the transfer section. The optimum values of the ESI-MS parameters were as follows: capillary voltage, 3.0 kV; sampling cone, 35.0 V; extraction cone, 4.0 V; source and desolvation temperature, 150 °C and 350 °C; cone and desolvation gas flow, 20.0 L/h and 600 L/h, respectively. During acquisition, leucine enkephalin was used as the mass reference (556.2771, M + H^+^), which was infused directly at a flow of 5 μL/min at a concentration of 2 ng/mL, allowing internal mass calibration. MS data were acquired in continuum mode and processed with MassLynx® (version 4.1, Waters®), and the compounds were putatively identified with Progenesis® QI for small molecules (Nonlinear Dynamics version 2.3, Waters®) using Chemspider and Progenesis MetaScope as identification methods. The search parameters were as follows: precursor tolerance of 30 ppm, theoretical fragmentation and fragment tolerance of 30 ppm, with an isotope similarity filter of 90%. The databases consulted were AraCyc, PlantCyc, KEGG and HMDB. Compounds such as gallic acid, methyl gallate, quercetin, quercitrin, myricetin, and fisetin were confirmed by comparing their retention times and MS/MS fragmentation patterns with analytical standards.

### Statistical analysis

The results are presented as the mean ± standard deviation (S.D.) of values obtained in three independent experiments performed in triplicate. The statistical analysis was conducted using one-way ANOVA for parametric data with a normal distribution, employing Minitab® software (version 16.1) and with Tukey’s and Dunnett’s tests for comparisons. The differences between the means among different treatments were considered to be significant when *p* ≤ 0.05.

## Results

### Physicochemical analysis of RHTR

Physicochemical parameters measured in RHTR stems are shown in Table 1; moisture and ash contents were within normal limits established by the WHO for the storage and use of medicinal plants [21]. The AE had more carbohydrates than other components such as proteins, amino acids or lipids. Differences in the concentration of primary metabolites among the analyzed samples were due to changes in the metabolic activity of RHTR, by its maturation process, regrowth or stress factors such as drought and temperature changes [42].

**Table 1:** Physicochemical analysis of RHTR as a quality control

Composition	Quantity
Carbohydrates	309.3 ± 27.5
Amino acids	15.9 ± 3.7
Proteins	21.1 ± 3.3
Lipids	4.5 × 10^−4^ ± 2.1 × 10^− 4^
% Humidity	7.9 ± 2.1
% Total ash	3.5 ± 0.04
% Soluble ash H_2_O	2.8 ± 0.5
% Insoluble ash HCl	1.0 ± 0.5
pH	4.4 ± 0.3

The results (mg/g) show the mean ± S.D. of three biological replicates (*n* = 3, in triplicate)

### Contents of polyphenols and antioxidants in AE and FF from RHTR

It has been reported in the literature, at least for *R. verniciflua*, *R. succedanea* and *R. coriaria*, that these species contain a high concentration of phenolic compounds, such as gallates and flavonoids with antioxidant, anti-inflammatory, antibacterial, antiparasitic, and antitumoral activities. [13]. Therefore, the AE-RHTR was fractionated to concentrate these compounds. The AE showed the highest concentration of polyphenols (94.14 ± 7.8 mg/g GAE), flavonoids (84.56 ± 3.6 mg/g QE), anthocyanins (9.98 ± 1.5 mg/g Cyd-3-Glu-E) and antioxidants (197.5 ± 11.3 mg/g TE, very high activity) compared to FF (Table 2). Additionally, AE showed a DPPH inhibition of 86.07% (highest activity) in contrast to the low activity of FF with only 14.10% inhibition; AEI could not be calculated because the samples did not reach the AC_50_ needed to inhibit only half of DPPH in the formula (Table 2). These results are apparently related to the yield or efficiency obtained from the fractionation of RHTR, whereby the FF presented lower concentrations than those in the AE. However, the values obtained in AE-RHTR were similar to those reported for green tea [43] and other *Rhus* sp. [12], which have been characterized as having high concentrations of these compounds. Carcinogenesis studies in cell lines and animal models have shown that plant extracts with abundant polyphenols can inhibit tumorigenesis during the initiation, promotion and progression stages through their anti/pro-oxidant effects [44], whereby the biological activity of RHTR was evaluated.

**Table 2:** Contents of polyphenols and antioxidants in RHTR

Samples	AE	FF
Weight (mg)	590 ± 49.5	22 ± 4.9
Polyphenols (Ga Eq)	94.14 ± 7.8	16.38 ± 3.8
Flavonoids (Que Eq)	84.56 ± 3.6	27.4 ± 5.7
Anthocyanins (Cya Eq)	9.98 ± 1.5	0.043 ± 0.01
Antioxidants (Trolox Eq)	197.5 ± 11.3	13.2 ± 0.09
AC_50_	0.042	0.105
T_AC50_	1	41
% DPPH Inhibition	86.07	14.10
AEI	ND	ND
Antioxidant activity	Very high	Low

The results (mg/g) show the mean ± S.D. of three biological replicates (*n* = 3, in triplicate). *DPPH* 2,2 diphenyl-1-picrylhydrazyl, *ND* not determined, *Ga* gallic acid, *Que.* quercetin, *Cya* cyanidin-3-glucoside, *Eq* equivalents, *AC*_*50*_ half antioxidant concentration (mg/mL), *T*_*AC50*_ time in which AC_50_ was observed (min), *AEI* antioxidant efficient index, *AE* aqueous extract, *FF* flavonoid fraction

### Biological activity of AE and FF from RHTR in cancer and normal cells

**Fig. 2:**
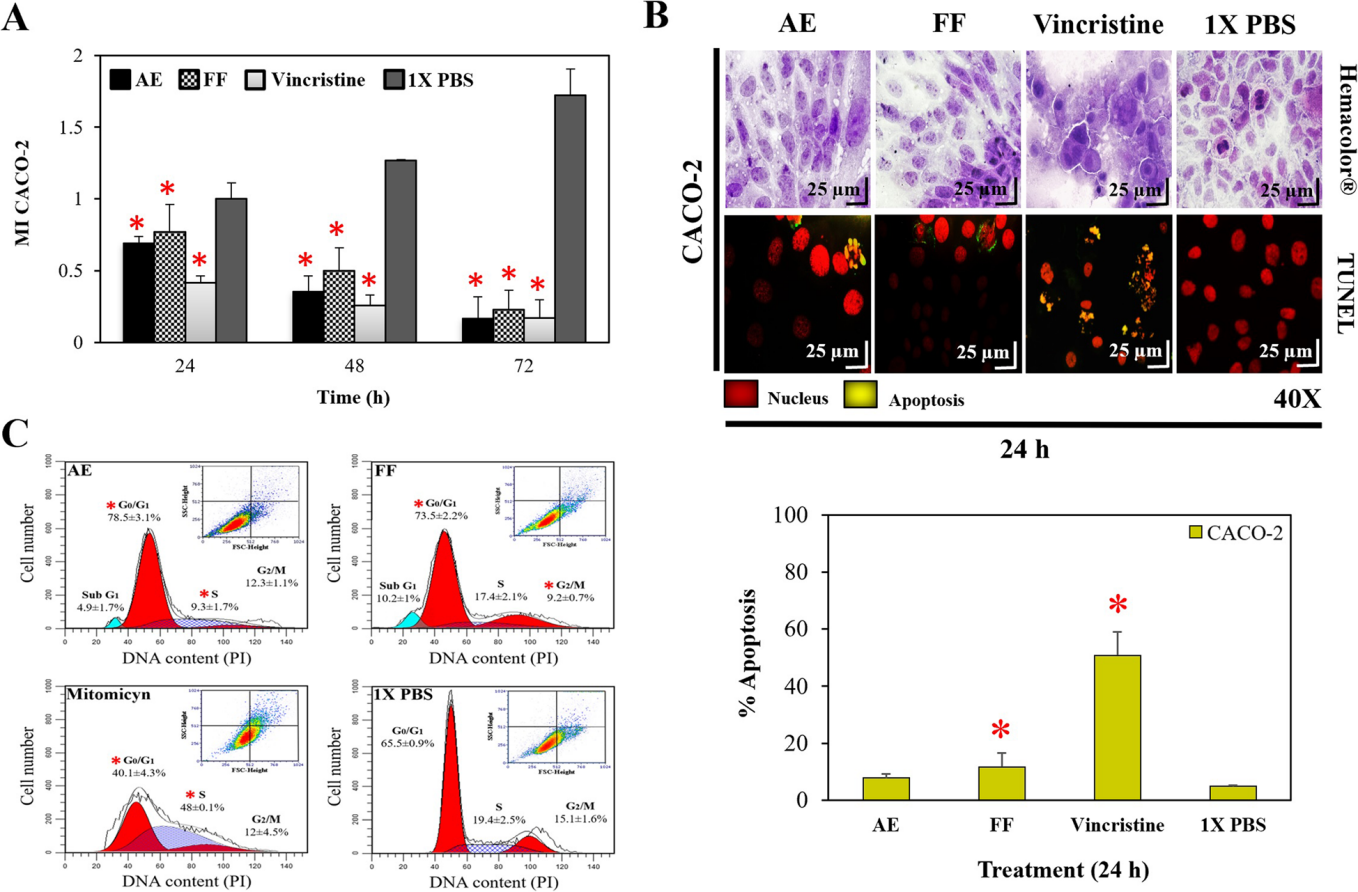
Biological activity of RHTR in colon cancer cells. The antiproliferative effects of AE and FF from RHTR were evaluated from 24 to 72 h with the IC_50_ previously determined by the MTT assay (**a**). The morphological changes and induction of apoptosis were determined after 24 h of treatment with AE (5 μg/mL) and FF (18 μg/mL) by Hemacolor® rapid staining and the TUNEL assay, respectively (**b**). The DNA content in different phases of the cell cycle during treatments with RHTR was determined at 24 h under the same conditions mentioned above by flow cytometry with PI (**c**). The results show the mean ± S.D. of three biological replicates (*n* = 3, in triplicates); *, *p <* 0.05 vs the control group without treatment (1X PBS)

**Fig. 3:**
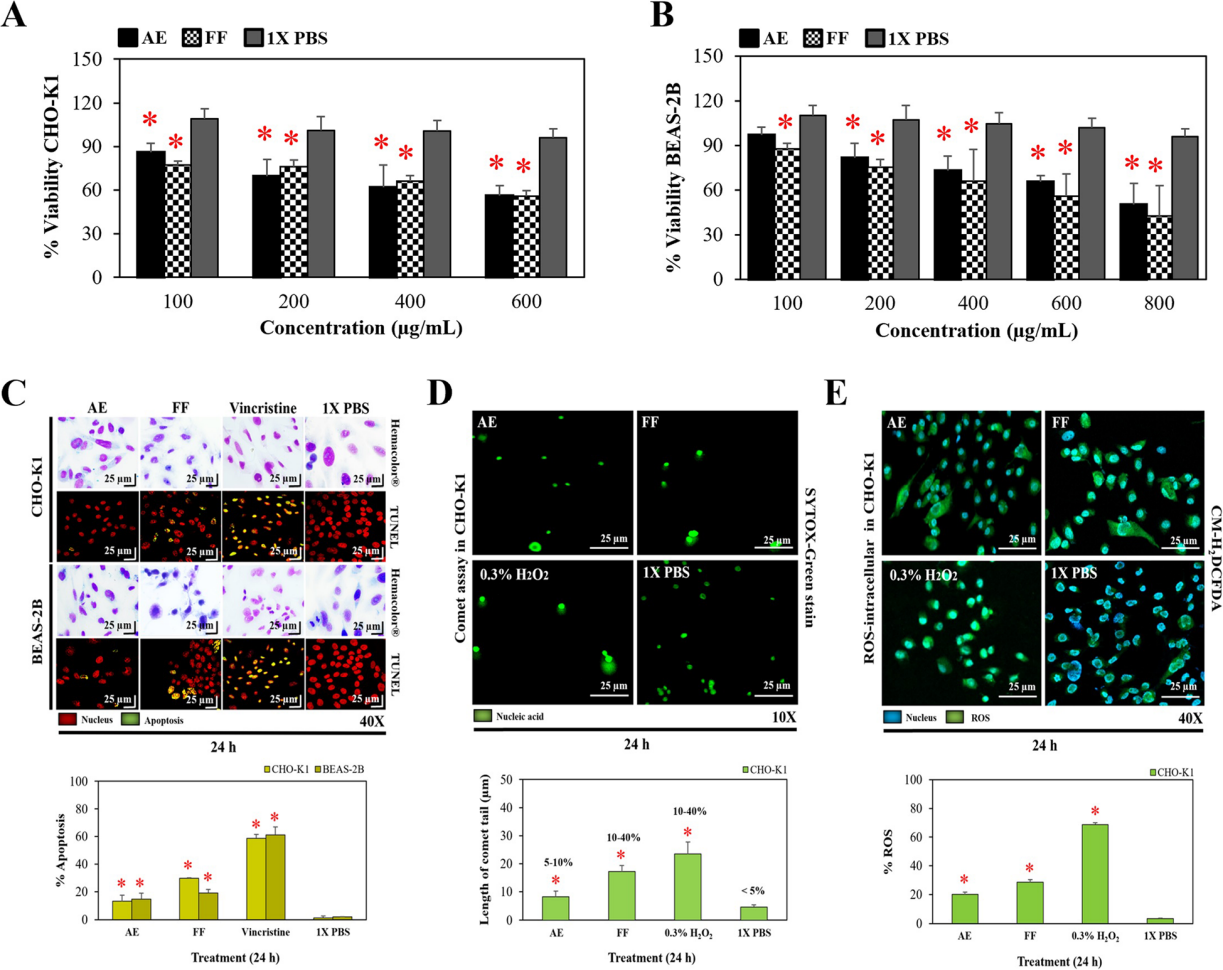
Biological activity of RHTR in normal cell lines. The toxicological effects of AE and FF from RHTR in CHO-K1 (**a**) and BEAS-2B (**b**) cells were evaluated using dose-response viability curves for 24 h by the MTT assay. The morphological changes with Hemacolor® rapid staining and induction of apoptosis by TUNEL assay were observed after 24 h of treatment with the IC_50_ of AE and FF (**c**). The migration of damaged DNA was determined by single cell gel electrophoresis under alkaline conditions (comet assay) after treatment for 24 h with AE and FF at 600 μg/mL in CHO-K1 cells (**d**). The production of intracellular ROS in CHO-K1 cells was determined by CM-H_2_DCFDA assays under the same conditions mentioned above (**e**). The results show the mean ± S.D. of three biological replicates (*n* = 3, in triplicates); *, *p <* 0.05 vs the control group without treatment (1X PBS)

Several studies have linked the anti/pro-oxidant activity of plants with their antineoplastic effect. Consequently, in this study the biological activity of RHTR was evaluated against colon adenocarcinoma cells (CACO-2). The IC_50_ of treatments with AE and FF at 24 h in CACO-2 cells was 5 and 18 μg/mL, respectively (*p* ≤ 0.05, Dunnett) (Table 3). Both samples, demonstrated biological activity below 100 μg/mL in accordance with the U.S. National Cancer Institute (NCI) guidelines for new therapeutic candidates against cancer [31, 45]. Subsequently, the IC_50_ doses calculated previously were used to evaluate their anti-proliferative activity in assays from 24 to 72 h in CACO-2 cells (Fig. 2a). The statistical analysis showed that all treatments were effective compared with cells treated with 1X PBS at different times (*p* ≤ 0.05, Dunnett). Moreover, AE and FF showed no differences from the vincristine dose formerly determined (IC_50_: 25 μg/mL, Table 3) (*p* > 0.05, ANOVA). The morphology and apoptosis assays used to expose cellular damage in CACO-2 revealed that cells treated with AE and FF had a typical morphology with respect to the control group (1X PBS), but with a considerable increase in cytoplasmic vesicles and absence of mitotic division, suggesting a quiescent/cytostatic effect (Fig. 2b). In addition, the TUNEL assay demonstrated nuclear DNA fragmentation in 7.85 ± 1.4% of the cells treated with AE and 11.6 ± 4.8% of the cells treated with FF due to the activation of an apoptotic process (*p* ≤ 0.05, ANOVA) (Fig. 2b). The determination of the cell cycle during each treatment with AE and FF showed 78.5 ± 3.1% and 73.5 ± 2.2% arrest at G_1_ phase, respectively (*p* ≤ 0.05, ANOVA), as well as the presence of a sub-G_1_ population of 4.9 ± 1.7% and 10.2 ± 1% related to the apoptotic processes (Fig. 2c). In dot-plot graphs, an increase in cellular granularity was observed for all treatments with respect to the 1X PBS control group (Fig. 2c). These results correlated and demonstrated that the main effect of RHTR was the arrest of the cell cycle and the subsequent induction of cell death by apoptosis in cancer cells. The in vitro toxicological assays in ovarian (CHO-K1) and lung/bronchus (BEAS-2B) epithelial cells revealed that AE had a lethal effect at 600/800 μg/mL respectively, while FF was toxic at 600 μg/mL (Table 3), reducing the cellular viability compared with the control group (*p* ≤ 0.05, Dunnett) (Fig. 3a and b). Morphological and TUNEL analyses demonstrated changes in the cells as a consequence of the administration of AE and FF at the above mentioned concentrations (Fig. 3c), among which a decrease in cellular cytoplasm, condensation of chromatin, loss of cellular contact, and absence of mitotic division were evident, as well as nuclear DNA fragmentation in 13 ± 4.3 / 14.7 ± 4.1% of the cells treated with AE and 29.6 ± 0.6 / 19.1 ± 2.4% of the cells treated with FF (*p* ≤ 0.05, ANOVA), respectively; all these alterations together demonstrated cell death by apoptosis. The comet assay and ROS determination in CHO-K1 cells showed that treatments with AE and FF induced a genotoxic effect that may have been due to the production of intracellular ROS; with DNA damage of 8.2 ± 1.9% (low effect) and 17.1 ± 2.1% (medium effect) according to the comet tail length, and ROS of 19.9 ± 1.6% and 28.5 ± 1.9%, respectively (*p* ≤ 0.05, ANOVA) (Fig. 3d and e). These results correlate with a highly selective effect of RHTR observed on cancer cells vs normal cells because AE had an SI of 144 (very high), while the FF was 33.3 (high) compared with that of the vincristine control, which had a low SI of 1.6 (Table 3), revealing that RHTR could become an important tool for the prevention and elimination of cancer; however, more in-depth studies are needed to support this hypothesis.

**Table 3:** Biological activity of RHTR in cell lines and *BALB/c* mice

	AE	FF	Vincristine	IX PBS
*Cells*	In vitro studies: IC_50_, SI and LD_50_			
IC_50_ CACO-2	5 (High)	18 (High)	25 (High)	> 800 (Very low)
IC_50_ BEAS-2B	800 (Very low)	600 (Very low)	25 (High)	> 800 (Very low)
IC_50_ CHO-K1	600 (Very low)	600 (Very low)	57 (medium)	> 800 (Very low)
SI	140 +++	33.3 ++	1.64 +	ND
LD_50_ [Theoretical]	1141.5	1209	350	ND
	In vivo studies: acute toxicity			
Sign toxicity	None	None	ND	None
Survivors (%)	100	100	ND	100
Final weight (g)	28.6 ± 2.4	28.2 ± 2.4	ND	26.5 ± 2.3

The results show the mean ± S.D. of three biological replicates (n = 3, in triplicate)

The values in parentheses correspond to the biological activity of the samples

Selective index (SI) indicate specific activity on cancer cells vs normal cells (+++, very high; ++, high; +, low)

*ND* not determined, *IC*_*50*_ half-maximal inhibitory concentration (μg/mL), *LD*_*50*_ median lethal dose (oral, mg/kg), *AE* aqueous extract, *FF* flavonoid fraction

### Acute toxicity evaluation in *BALB/c* mice with AE and FF from RHTR

**Fig. 4:**
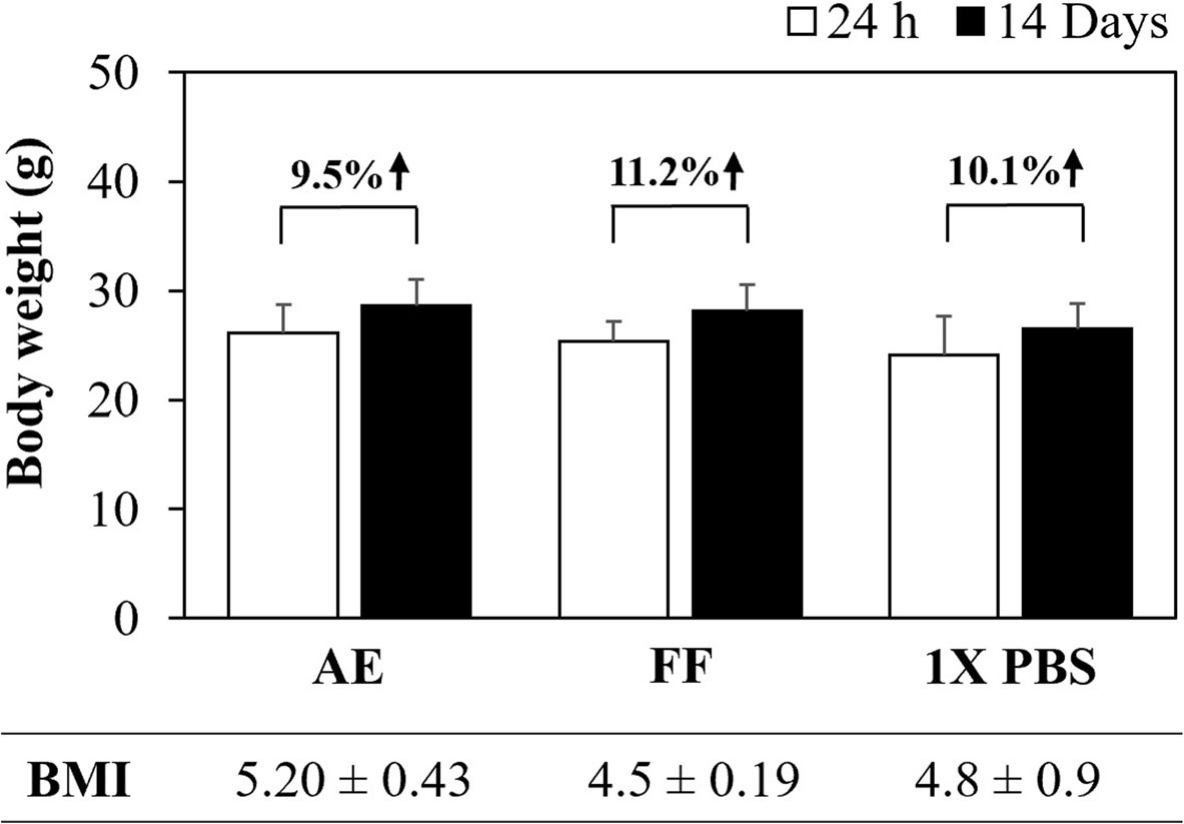
Body weight evaluation of *BALB/c* mice treated with RHTR. Effect of AE and FF from RHTR at 200 mg/kg on the body weight of mice at 24 h and 14 days posttreatment, where percentages indicate weight gain. The table shows the body mass index (BMI). The results show the mean ± S.D. of two biological replicates (*n* = 5); *, *p* < 0.05 vs the control group without treatment (1X PBS)

**Fig. 5:**
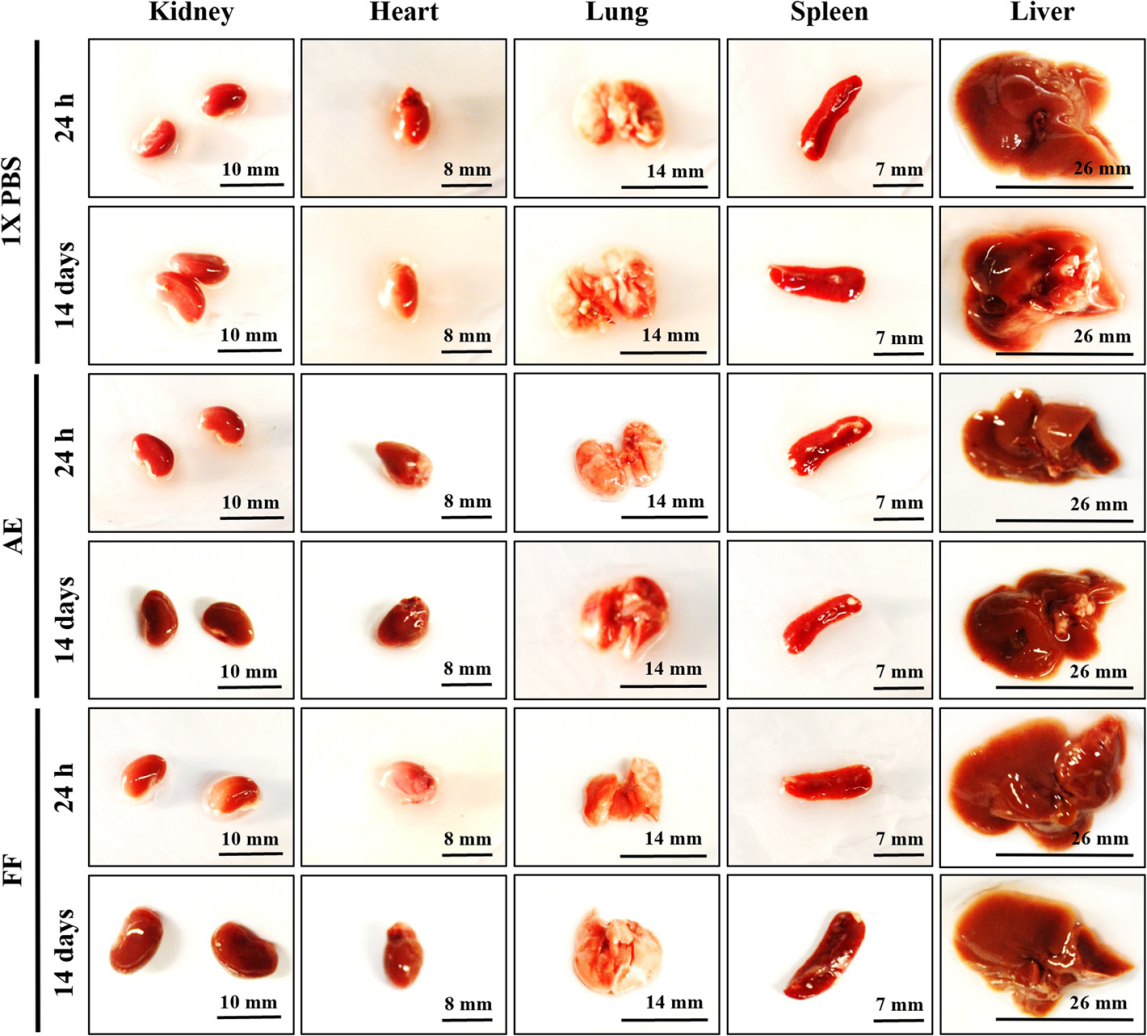
Morphological analyses of organs extracted from necropsy of *BALB/c* mice treated with RHTR. Anatomical observations of the main organs extracted in the laparotomy were performed. The results are representative of two biological replicates (*n* = 5)

**Fig. 6:**
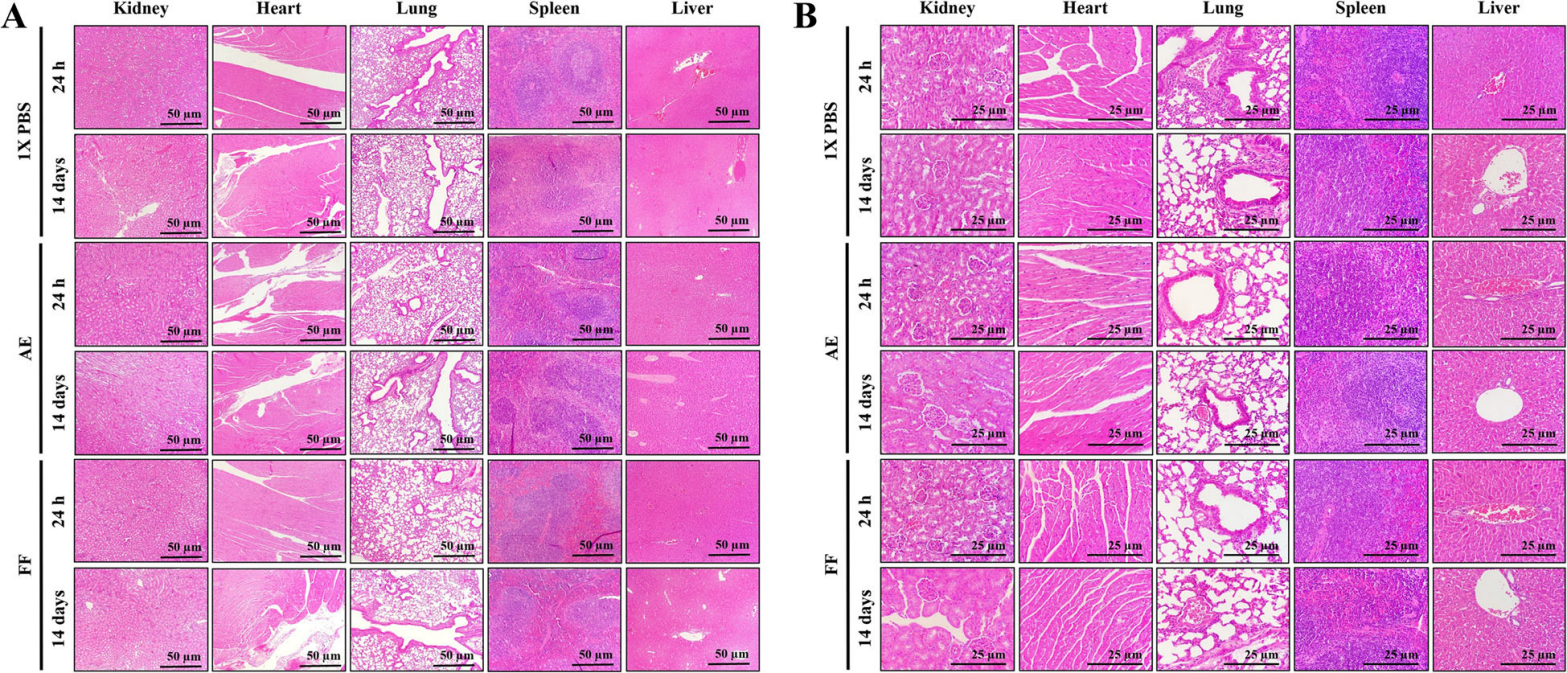
Histological analyses of organs obtained from mid-laparotomy of *BALB/c* mice treated with RHTR. Representative sections of mouse kidney, heart, lung, spleen and liver shown at 10X (**a**) and 40X (**b**) magnifications. The results are representative of two biological replicates (*n* = 5)

After i.p. administration of AE and FF at 200 mg/kg using the “up-and-down” method, no behavioral changes (agitation, tremor, drowsiness, loss of appetite) or signs of toxicity (dyspnea, photophobia, blindness, diarrhea, heart failure, muscle weakness, seizures, epithelial pigmentation) were observed in rodents during the initial hours or after 24 h of treatment. The assay was extended for 14 days to determine the number of surviving animals, which was 100% in both cases and to calculate the LD_50_ for AE (1141 mg/kg) and for FF (1209 mg/kg), respectively (Table 3). The mice did not present significant changes in body weight compared with the control group (*p* ≤ 0.05, ANOVA), resulting in a weight gain and BMI of 9.5% (5.20 ± 0.43) for AE and 11.2% (4.5 ± 0.19) for FF, with the latter being higher than the control group, which was 10.1% (4.8 ± 0.9) (Fig. 4). Anatomical morphologies were consistent with the control group that received vehicle as treatment (Fig. 5). The morphometric analysis (Table 4) revealed significant differences only in the diameter of the heart (8.8 ± 0.45 mm) in mice exposed to AE for 24 h and in the weight of the spleen after 24 h (0.10 ± 0.03 g) or 14 days (0.14 ± 0.01 g) of treatment with FF (*p* ≤ 0.05, ANOVA). However, the changes observed in these organs did not present a % ROW lower or higher than 10% with respect to the control group; thus, these changes can be considered unrelated to pathological causes [41]. Subsequently, histological sections of organs were prepared to search for differences in cellular morphology compared with the control group (Fig. 6a and b). In all cases, control as well as treated animal organs displayed a normal appearance. In spleen, at 10X magnification, capsules with trabeculae were observed, and the splenic parenchyma consisted of white and red pulp; at 40X magnification, a Malpighian corpuscle was observed, in which the central artery surrounded by the germinal center and lymphocytes were distinguished. At 10X magnification, the kidney exhibited its typical external crust and internal medulla, while at 40X magnification, numerous collecting tubes with a rectilinear path and renal glomeruli surrounded by Bowman’s capsule were observed. At 10X magnification, the lungs showed the visceral leaf of the pleura surrounding the pulmonary parenchyma with some bronchi and bronchioles, as well as blood vessels of different caliber and empty spaces corresponding to alveolar sacs, while at 40X, the bronchial wall and muscular layer were observed. At 10X, the heart showed numerous striated muscle fibers surrounded by connective tissue with abundant blood vessels, while at 40X, cardiac muscle fibers with a central nucleus and intercalary discs were observed. In the liver, the portal triad composed of a portal vein, the hepatic artery and a biliary canal, were clearly observed at both magnifications. Based on these results, histological lesions were ruled out during the RHTR treatments. Finally, a hematological analysis revealed slight leukopenia in mice treated with the AE at 14 days after administration and with the FF during the different evaluation times, as well as mild erythropenia compared with the control group (*p* ≤ 0.05, ANOVA) or reference values (Table 5), while the biochemical analysis demonstrated an elevation in the AST for AE and ALT for FF at 24 h after administration (*p* ≤ 0.05, ANOVA). Hence, other parameters such as total proteins and albumin were considered to rule out the deterioration of hepatic function, which were within the normal reference range for mice. Triglycerides were slightly elevated after 14 days of starting treatment with AE with respect to the control group, but the above mentioned values were also maintained within the normal reference range for mice (Table 5). These results suggest a faint suppression of hematopoiesis in the bone marrow during the administration of the AE and FF from RHTR due to the highly replicative phenotype of the blood cells [46]; however, the biochemical parameters did not reveal hepatic dysfunction or renal impairment, and therefore, the use of RHTR may be considered as an alternative cancer treatment.

**Table 4:** Morphometric analysis of organs in *BALB/c* mice treated with RHTR

Time	Treatment	Measurement	Kidney	Heart	Lung	Spleen	Liver
24 h	AE	W	0.36 ± 0.04 (1.4%)	0.13 ± 0.02 (0.5%)	0.22 ± 0.02 (0.8%)	0.11 ± 0.01 (0.4%)	1.44 ± 0.2 (5.5%)
		D	9.23 ± 0.5	8.8 ± 0.45 *	11.04 ± 1.4	14.5 ± 1.16	29.6 ± 0.96
	FF	W	0.37 ± 0.04 (1.5%)	0.12 ± 0.01 (0.5%)	0.22 ± 0.05 (0.9%)	0.10 ± 0.03 * (0.4%)	1.36 ± 0.3 (5.4%)
		D	10.17 ± 0.8	8.42 ± 0.64	11.6 ± 0.42	15.1 ± 1.8	26.1 ± 2.96
	1X PBS	W	0.35 ± 0.06 (1.4%)	0.12 ± 0.02 (0.5%)	0.23 ± 0.03 (0.9%)	0.13 ± 0.02 (0.5%)	1.3 ± 0.21 (5.4%)
		D	10.06 ± 0.5	8.19 ± 1.2	11.94 ± 1.1	14.1 ± 2.12	27.9 ± 1.2
14 days	AE	W	0.37 ± 0.01 (1.3%)	0.16 ± 0.02 (0.5%)	0.26 ± 0.02 (0.9%)	0.11 ± 0.01 (0.4%)	1.29 ± 0.09 (4.5%)
		D	10.3 ± 0.55	9.5 ± 1.7	13.74 ± 0.9	17.4 ± 1.15	28.6 ± 4.83
	FF	W	0.22 ± 0.14 (0.8%)	0.17 ± 0.02 (0.6%)	0.28 ± 0.05 (0.9%)	0.14 ± 0.01 * (0.5%)	1.48 ± 0.15 (5.3%)
		D	10.34 ± 0.4	9.35 ± 0.14	13.93 ± 1.7	17.01 ± 0.6	30.5 ± 0.86
	1X PBS	W	0.35 ± 0.05 (1.3%)	0.16 ± 0.03 (0.6%)	0.21 ± 0.01 (0.8%)	0.12 ± 0.01 (0.5%)	1.35 ± 0.12 (5.1%)
		D	10.8 ± 0.6	10.03 ± 0.8	14.6 ± 2.95	15.2 ± 1.4	31.8 ± 3.3

The results show the mean ± S.D. of two biological replicates (*n* = 5)

Measurement of W: weight (g), D: larger diameter (mm) and (% ROW): relative organ weight, for each organ

*, *p* < 0.05 vs control values without treatment (1X PBS)

*AE* aqueous extract, *FF* flavonoid fraction

**Table 5:** Effect of RHTR on biochemical and hematological parameters of *BALB/c* mice in the acute toxicity study

Time	24 h.			14 days			Reference range
Treatment	AE	FF	1X PBS	AE	FF	1X PBS	(Midrange)
BIOCHEMICAL PARAMETERS							
Glucose (mg/dL)	118.7 ± 13.8	113.7 ± 17.5	138.7 ± 17.9	130.2 ± 6.9	127.7 ± 15.4	141.5 ± 10.4	63–176 (89)
Triglycerides (mg/dL)	70.13 ± 11.3	74.6 ± 7.4	81.4 ± 8.6	**121.8 ± 17.2**	112.75 ± 19.8	98.5 ± 7.3	55–115 (85)
Cholesterol (mg/dL)	76.3 ± 8.5	77.5 ± 14.4	75 ± 8.2	61 ± 12.7	55 ± 5.8	65 ± 2.8	26–82 (64)
Protein (g/dL)	5.4 ± 0.5	5.5 ± 0.6	5.4 ± 0.25	5.7 ± 0.63	5.2 ± 0.5	5.8 ± 0.82	4–8.6 (6.2)
AST (TGO) (U/L)	205 ± 45.3 *	152.5 ± 8.7	127.5 ± 19.4	132 ± 19.7	165 ± 45.5	161.8 ± 47.5	55–251 (139)
ALT (TGP) (U/L)	55.9 ± 8.8	78.7 ± 0.3 *	52.9 ± 6.2	64.4 ± 1.4	74.7 ± 16.1	70.7 ± 5.8	17–77 (47)
LDH (U/L)	**1234 ± 221**	**954 ± 281**	**1330 ± 274**	**1122 ± 201**	**980 ± 286**	**997.3 ± 70.3**	149–215 (182)
Creatinine (mg/dL)	0.62 ± 0.2	0.5 ± 0.01	0.5 ± 0.01	0.5 ± 0.01	0.5 ± 0.01	0.46 ± 0.09	0.2–0.9 (0.5)
Urea (mg/dL)	60 ± 5.8	56.2 ± 4.8	53.7 ± 6.3	65 ± 4.1	57.5 ± 6.5	56.7 ± 7.2	46.9–73 (60.1)
HEMATOLOGICAL PARAMETERS							
Hemoglobin (g/dL)	15.16 ± 1.09	13.5 ± 0.36	14.76 ± 0.90	16 ± 1.05	13.23 ± 1.8	14.13 ± 0.20	10–17 (13.1)
Hematocrit (%)	47.2 ± 4.07	39.33 ± 3.40	43.93 ± 2.83	49.5 ± 3.99	38.8 ± 5.63	43.7 ± 0.55	39–49 (40.4)
Erythrocytes (X10^6^/mm^3^)	9.69 ± 1.16	**7.34 ± 0.95**	9.27 ± 0.97	9.92 ± 0.85	**7.91 ± 1.13**	8.84 ± 0.43	8.3
MCHC (%)	32.06 ± 0.66	**36.36 ± 4.56**	33.63 ± 1.04	32.3 ± 0.52	**36.26 ± 2.57**	32.3 ± 0.72	32.3
MCH (pg)	15.66 ± 0.8	**14.63 ± 3.68**	15.96 ± 0.96	16.1 ± 0.34	**14.3 ± 2.4**	15.96 ± 0.86	15.9
MCV (μL)	48.9 ± 1.6	47.26 ± 11.49	47.5 ± 1.83	49.9 ± 0.3	48.9 ± 1.95	49.5 ± 1.83	49.1
Leukocytes (× 100/mm^3^)	5000 ± 953.9	**4233.3 ± 1404.7**	5666.66 ± 808.29	**4466.66 ± 929.15**	**4666.6 ± 1242.3**	5033.33 ± 1227.8	5–12 (6.33)
RDW-CV	15 ± 1.17	18.7 ± 1.56	17.96 ± 1.11	13.73 ± 0.57 *	16.33 ± 0.97	16.16 ± 1.20	
RDW-SD	24.26 ± 3.18 *	32.63 ± 18.9	33.2 ± 5.32	22.8 ± 0.51*	26.13 ± 1.5	25.73 ± 1.38	
Platelets (/μL)	**437,333 ± 94,214**	**558,666.6 ± 230,048.5**	**627,000 ± 95,859.2**	**368,000 ± 46,130**	**425,333.3 ± 325,423**	**572,666 ± 158,190**	116 × 10^3^
MPV (fL)	7.6 ± 0.2	7.5 ± 2.4	8.16 ± 0.87	7.56 ± 0.15	9.43 ± 0.49 *	7.6 ± 0.3	
PDW (%)	14 ± 0.1	14.6 ± 4.5	14.96 ± 2.32	14.06 ± 0.15	13.03 ± 2.8	14 ± 0.26	
PCT (%)	0.33 ± 0.07 *	0.27 ± 0.05 *	0.51 ± 0.12	0.277 ± 0.03 *	0.585 ± 0.12	0.432 ± 0.10	
Lymphocytes (%)	67.33 ± 10.06 *	74 ± 7.21	82 ± 2	65 ± 13.4	63.3 ± 4.16	68 ± 20.7	35–90
Monocytes (%)	1.33 ± 1.15	0	0.33 ± 0.57	0.33 ± 0.57	0	0	0–3
Eosinophils (%)	2	0	0	0	0	0	0–7
Basophils (%)	0	0	0	0	0	0	0–1
Segmented neutrophils (%)	38 ± 9.16 *	25.3 ± 7.5	17.66 ± 2.08	34.66 ± 13.61	16.66 ± 4.16	24.66 ± 27.30	10–40
Neutrophils band (%)	0	0	0	0	0	0	0
Immature shape (%)	0	0	0	0	0	0	0

The results show the mean ± S.D. of two biological replicates (*n* = 5)

The numbers in bold indicate differences with respect to reference values for the mouse

*, *p* < 0.05 vs the 1X PBS control group (without treatment)

References range, minimum and maximum normal value for the analyte of interest in *BALB/c* mice and the respective midrange

*AE* aqueous extract, *FF* flavonoid fraction

*AST (TGO)* aspartate aminotransferase, *ALT (TGO)* alanine aminotransferase, *LDH* lactate dehydrogenase, *MCHC* mean corpuscular hemoglobin concentration, *MCH* mean corpuscular hemoglobin, *MCV* mean corpuscular volume, *RDW-CV* red blood cell distribution width as coefficient of variation, *RDW-SD* red blood cell distribution width as standard deviation, *MPV* mean platelet volume in femtoliters, *PDW* platelet distribution width, *PCT* platelecrit

### Phytochemical characterization of AE and FF from RHTR

**Fig. 7:**
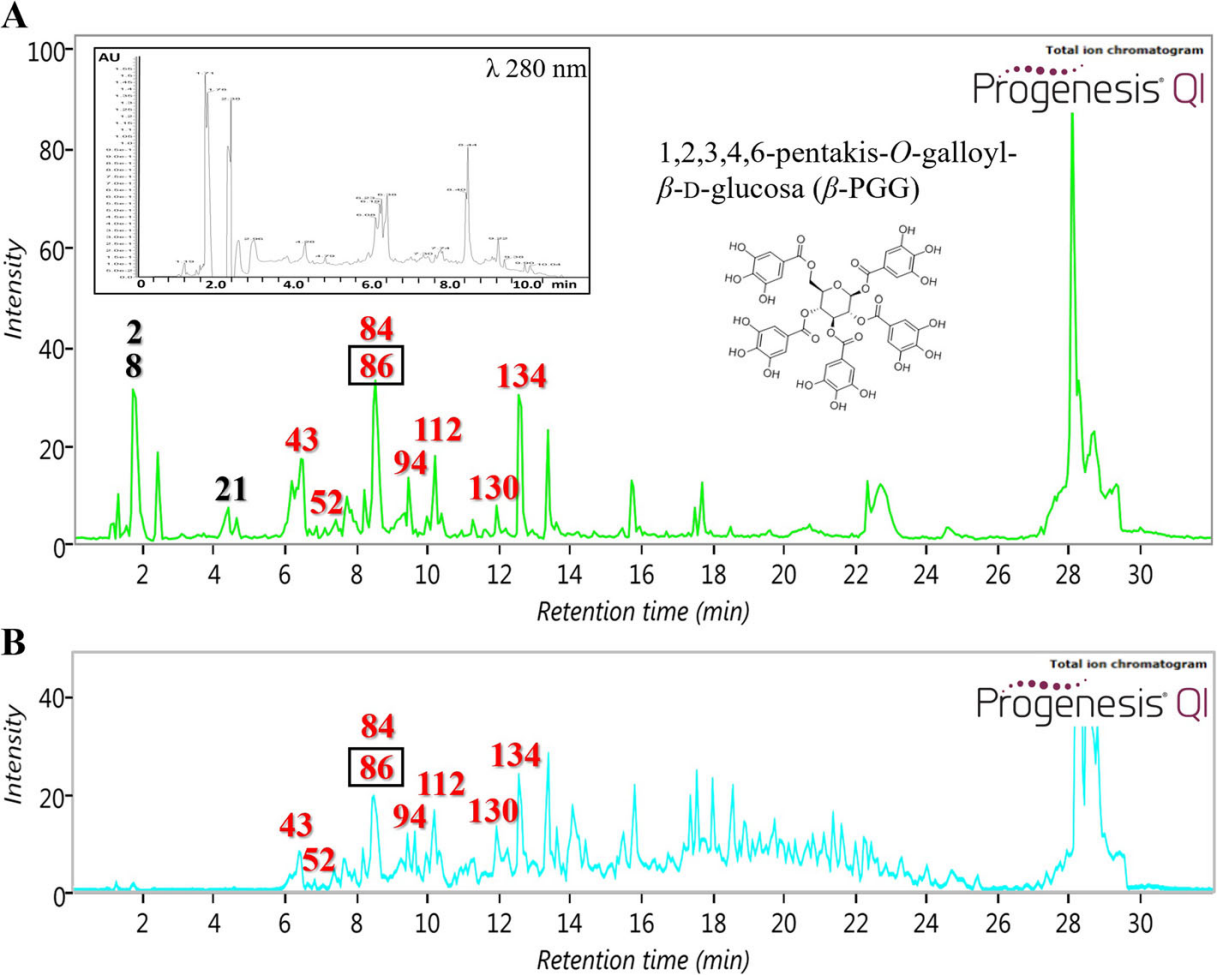
Chromatographic profile of RHTR by UPLC-MS^E^. TIC on ESI^+^ of AE (**a**) and FF (**b**) from RHTR showing the metabolic profiles of both samples with 173 preidentifications. The main compounds are indicated by the number of the corresponding order of appearance in Additional file 3: Table S1. The most abundant compounds in AE and FF are marked in red. The compound with higher relative abundance was identified, and a fingerprint of AE-RHTR by UPLC-PAD analysis at λ 280 nm is indicated in the corresponding inset. The results are representative of three biological replicates (*n* = 3, in triplicate)

In RHTR, 173 compounds among 272 features present in AE were putatively identified by comparison with databases for plants (Additional file 3: Table S1). The UV profile at 280 nm and TIC of AE-RHTR showed that the major classes of compounds were flavonoids (29%), followed by fatty acids (22%), phenolic acids (11%) and glycosylated compounds (10%) (Fig. 7a). The most abundant compounds in AE-RHTR were galactinol (2), 1-*O*-galloyl-*β*-D-glucose (8), quinic acid (21), methyl gallate (43), epigallocatechin 3-cinnamate (52), quercetin 3-(2″‘-galloylglucosyl)-(1 → 2)-alpha-L-arabinofuranoside (84), 1,2,3,4,6-pentakis-*O*-galloyl-*β*-D-glucose (*β*-PGG, 86), 4-*O*-digalloyl-1,2,3,6-tetra-*O*-*β*-D-galloylglucose (94), myricetin 3-(4″-galloylrhamnoside) (112), fisetin (130) and margaric acid (134) (Additional file 3: Table S1, Fig. 7a), which were identified by the fragmentation patterns of each compound and by matching their retention times with analytical standards (Additional file 2: Figure S2); all of these compounds present diverse activities in plants as primary or secondary metabolites. However, comparing the metabolite profiles of AE and FF showed that 43 (RT, 6.44), 52 (RT, 7.05), 84, 86 (RT, 8.52), 94 (RT, 9.13), 112 (RT, 9.97), 130 (RT, 11.92) and 134 (RT, 12.54) maintained a high relative abundance in both samples (Additional file 3: Table S1, Fig. 7b). These compounds have been reported to possess cytotoxic activity in cancer cells [13, 44, 47] and may be related to the medicinal properties of RHTR observed in this study (Additional file 3: Table S1). However, complementary studies are required to demonstrate whether there is a major compound in FF or a responsible synergistic mechanism. Additionally, urushiol, a toxic compound present in other *Rhus* sp., was not detected in AE and FF. Studies in RHTR have reported that gallic acid and ethyl gallate are among the main bioactive compounds in plants [10]. In our study, gallic acid (80) was found in AE and FF, whereas ethyl gallate was not present. However, 43 (RT, 6.44) was detected as mentioned above (Additional file 3: Table S1). The presence of gallic acid and methyl gallate in the extracts was derived from catabolic reactions of *β*-PGG, which is used by RHTR to elaborate new secondary metabolites against different stressors [48, 49]. Recent studies have shown that medicinal plants that are thermally processed by decoction or infusion, compared with other forms of preparation, have increased biological activity caused by chemical changes during heat treatment [50].

## Discussion

Plants can produce secondary metabolites, such as alkaloids, terpenoids and polyphenols, among other compounds, which can play an important role in the prevention and treatment of various diseases [51]. In vitro studies with AE and FF from RHTR on different cell lines have shown that low concentrations of both samples have a selective inhibitory effect on CACO-2 cell proliferation through induction of a quiescent/cytostatic state and later activation of an apoptotic process, compared with BEAS-2B and CHO-K1 normal cells. Some studies have demonstrated that malignant cell populations may experience a state of persistent cytostasis due to intrinsic and exogenous cellular factors, such as DNA damage, alteration of cyclin-dependent kinases and components of the PI3K signaling pathway or production of oxidative environments, among others [52]. These conditions can be induced by polyphenolic compounds present in plants such as RHTR. Therefore, the metabolic profiles of AE and FF from RHTR were analyzed to preidentify the major compounds. The analysis revealed a high content of polyphenols and flavonoids with antioxidant activity in AE-RHTR, which included methyl gallate, epigallocatechin 3-cinnamate, quercetin 3-(2″‘-galloylglucosyl)-(1 → 2)-alpha-L-arabinofuranoside, *β*-PGG, 4-*O*-digalloyl-1, 2, 3, 6-tetra-*O*-*β*-D-galloylglucose, myricetin 3-(4″-galloylrhamnoside), and fisetin. The concentrations of these compounds were increased in the FF fraction, and other research groups have reported that some of these compounds are important active principles against cancer by activating different molecular mechanisms involved in the state of quiescence and cell death [13, 44, 47]. Thus, we considered that the antineoplastic activity observed in RHTR might be related to the combination of these compounds in the AE. However, complementary studies are required to demonstrate if there is a major compound in FF or a synergistic mechanism responsible for the medicinal properties of RHTR. Moreover, urushiol, a toxic compound present in other *Rhus* sp., was not detected in RHTR. The *Rhus* genus has been shown to have a high concentration of phenolic compounds, such as gallic acid (among other gallates), and flavonoids, such as fustin, myricetin, quercetin, butein and sulfuretin, with antioxidant, anti-inflammatory, antibacterial, antiparasitic and anticancer activities reported for *R. verniciflua*, *R. succedanea* and *R. coriaria*, respectively [13]. The most studied mechanism of polyphenols is their anti/pro-oxidant effect. Studies performed with green tea polyphenols (*Camellia sinensis*) [43, 53] have demonstrated that these compounds undergo auto-oxidative reactions resulting in the production of ROS, which play an important role in cells [44, 54]. Several authors have shown that ROS act as second messengers and modulate the activity of various processes related to the cytoskeleton, the cell cycle and cell death in cancer and normal cells, such as inhibition of *β*-transforming growth factor (TGF-*β*) and epidermal growth factor (EGF), or phosphorylation of histone 2A.X (*γ*H2A.X) (a marker of oxidative DNA damage), among others [55, 56]. Additionally, polyphenols have a selective effect on cancer cells because they exhibit a reduced regulation of the cellular machinery and, therefore, are more sensitive to the induction of quiescence and apoptosis, while normal cells can activate endogenous antioxidant mechanisms (e.g., glutathione *S*-transferase, *γ*-glutamyltransferase, and superoxide dismutase) to prevent cell death by DNA damage or lipid peroxidation [57, 58]. Therefore, this background could explain why the flavonoids in RHTR selectively induced cell cycle arrest in G_1_ and cell death via apoptosis at a lower concentration in CACO-2 but not in CHO-K1 and BEAS-2B cells. Moreover, studies have indicated that high doses of polyphenols have potential toxicity, possibly due to differences in the sensitivity, metabolism and bioavailability of polyphenols among individuals [44]. In humans, elevations of serum transaminase and bilirubin levels, abdominal pain, jaundice, portal/periportal inflammation and necrosis have been observed, all of which are symptoms related to hepatotoxicity [53, 59]. Therefore, toxicological assays with RHTR in the animal model were performed to determine possible adverse effects during its consumption. Regarding the toxicological study, the administration to rodents of 200 mg/kg of AE and FF from RHTR did not reveal behavioral changes or signs of toxicity. When performing necropsy for morphometric and histopathological analysis, no significant anatomical changes or histological lesions were found in the organs recovered from the different treatment groups with respect to the control group. However, hematological parameters revealed slight leukopenia in mice treated with AE and FF at 14 days posttreatment. Additionally, FF caused mild erythropenia compared with the control group and reference values. These results suggested a faint suppression of hematopoiesis in the bone marrow during administration of the AE and FF from RHTR, possibly due to the highly replicative phenotype of blood cells, which are more sensitive to the action of different cytotoxic compounds, as is often the case during the administration of chemotherapies in the treatment of cancer [46]. Our results regarding animal toxicity are similar to those found for *R. verniciflua*, for which the toxicity of fermented *Rhus verniciflua* stem bark extract (FRVSB, urushiol-free) was evaluated in *Sprague-Dawley* rats at a simple oral dose and at repeated doses of 5000 mg/kg body weight for 90 days, without observing the death of any rodent or adverse effects on clinical signs, body weight or food consumption [60]. When performing necropsy, the organs did not present anatomical or histological alterations during both tests, and analysis of biochemical and hematological parameters revealed no differences compared with the reference range for rats [60]. These results together with those discussed previously strongly suggest that, minimally, the AE and/or FF derived from RHTR could be administered without risk of affecting health and, most likely, without causing adverse effects as severe as those observed during the administration of conventional chemotherapy.

## Conclusions

The results obtained in this study demonstrated a selective antiproliferative activity of AE and FF from RHTR against CACO-2 colon cancer cells. This activity was found to be related to ROS generation that induced a quiescent state and later apoptotic process. Among the most abundant compounds were methyl gallate, epigallocatechin 3-cinnamate, quercetin 3-(2″‘-galloylglucosyl)-(1 → 2)-alpha-L-arabinofuranoside, *β*-PGG, 4-*O*-digalloyl-1,2,3,6-tetra-*O*-*β*-D-galloylglucose, myricetin 3-(4″-galloylrhamnoside), and fisetin. It has been demonstrated that these compounds can produce ROS and activate different molecular mechanisms involved in the state of quiescence and cell death; thus, the antineoplastic activity observed in RHTR might be related with these compounds. However, complementary studies are required to demonstrate this correlation. Moreover, these compounds could be considered as future prospects for more detailed studies. Furthermore, the results from the toxicological assay did not reveal significant anatomical changes or histological lesions in the organs recovered from the treatments with AE and FF, thus suggesting that RHTR might be nontoxic upon acute exposure during i.p. administration in mice. However, hematologic studies suggested that the RHTR decoction might have suppressive effects on hematopoietic processes if used at high concentrations for prolonged periods. Thus, it is suggested that this agent be used with caution. Finally, the high concentration of polyphenols and antioxidants in AE may have beneficial effects as chemoprotective agents against different degenerative diseases associated with oxidative stress. However, further studies are required to confirm this statement.

## Additional files

Additional file 1: **Figure S1.** Timeline scheme of the acute toxicity studies described in the experimental procedures. (PPTX 1372 kb)

Additional file 2: **Figure S2.** MS/MS analysis of the most abundant compounds in RHTR. (PPTX 1780 kb)

Additional file 3: **Table S1.** Phytochemical compounds putatively identified in RHTR by UPLC-MS^E^. (XLSX 37 kb)

## Data Availability

All data generated or analyzed during this study are included in this published article (as well as supplementary information files). Raw data are available from the corresponding author on reasonable request.

## Abbreviations

AC_50_: Half antioxidant concentration
ACs: Active compounds
AE: Aqueous extract
AEI: Antioxidant efficient index
AraCyc: Metabolic pathway databases for *Arabidopsis thaliana*
BMI: Body mass index
BWC: Body weight change
CM-H_2_DCFDA: (5-(and-6)-chloromethyl-2′,7′-dichlorodihydrofluorescein diacetate
DPPH: 2,2 diphenyl-1-picrylhydrazyl
EGF: Epidermal growth factor
ESI: Electrospray ionization source
EtOH: Ethanol
FBS: Fetal bovine serum
FF: Flavonoid fraction
HMDB: Human metabolome database
i.p: Intraperitoneal
IC_50_: Half-maximal inhibitory concentration
KEGG: Kyoto encyclopedia of genes and genomes
LD_50_: Median lethal dose
MeOH: Methanol
MI: Mitotic index
MS: Mass spectrometry
MTT: 3-(4,5-dimethylthiazol-2-yl)-2,5-diphenyltetrazolium bromide
PAD: Photodiode array detector
PlantCyc: Plant metabolic pathway databases
RHTR: *Rhus trilobata*
ROS: Reactive oxygen species
ROW: relative organ weight
SI: Selective index
TGF-*β*: Transforming growth factor beta
TIC: Total ion chromatogram
Trolox: 6-hydroxy-2,5,7,8-tetramethylchroman-2-carboxylic acid
UPLC: Ultra-performance liquid chromatography
UV: Ultraviolet
*β*-PGG: 1,2,3,4,6-pentakis-*O*-galloyl-*β*-D-glucose
