# Supplementary material for: Biological and toxicological evaluation of Rhus trilobata Nutt. (Anacardiaceae) used traditionally in mexico against cancer
Source: BMC Complement Altern Med. 2019 Jul 1;19:153. doi: 10.1186/s12906-019-2566-9 (PMC6604276; doi:10.1186/s12906-019-2566-9)
Supplement: Supplementary file 1 — Figure S1. Timeline scheme of the acute toxicity studies described in the experimental procedures. (PPTX 1372 kb) [file 12906_2019_2566_MOESM1_ESM.pptx]

## Slide 1
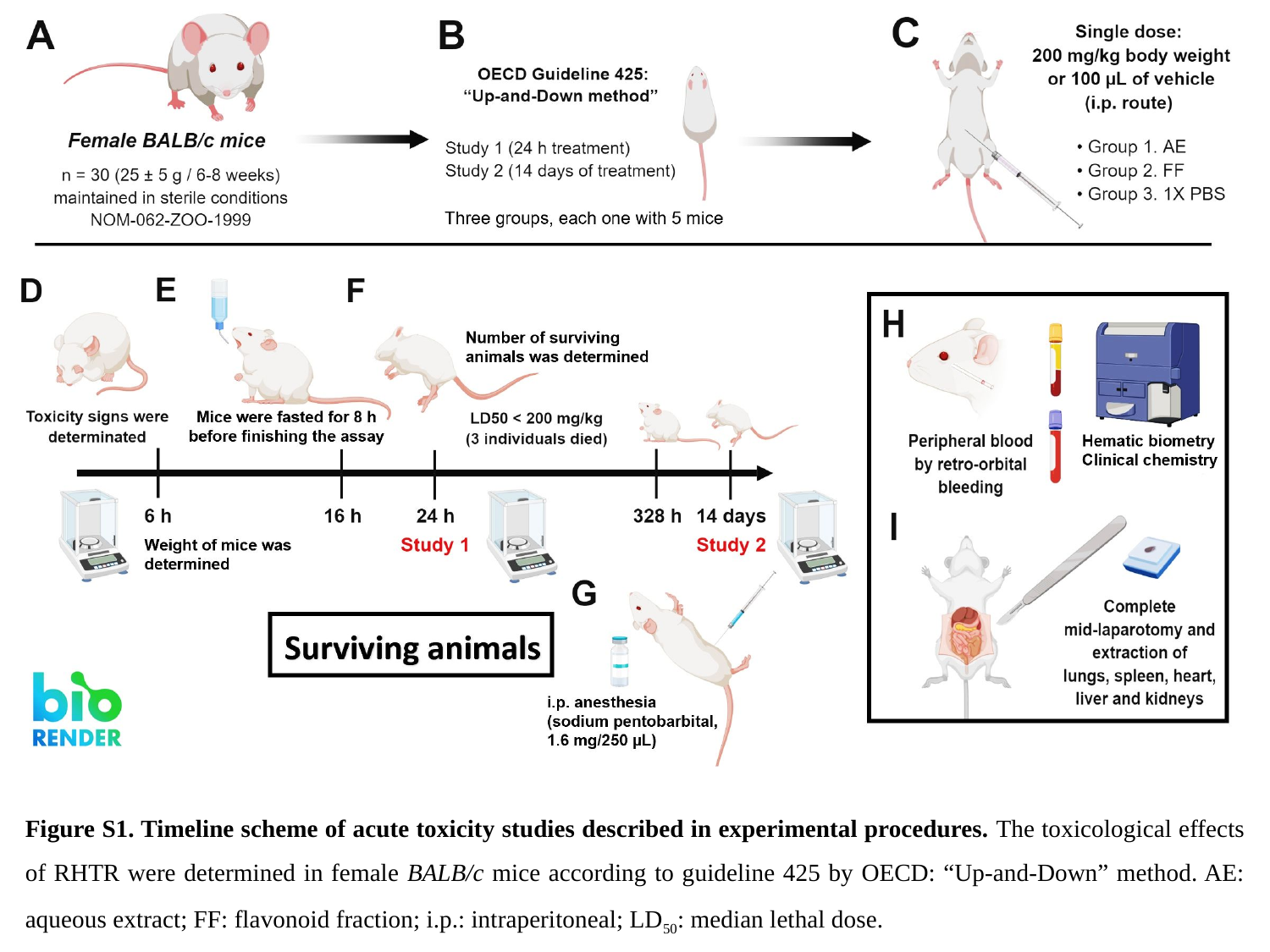

Figure S1. Timeline scheme of acute toxicity studies described in experimental procedures. The toxicological effects of RHTR were determined in female BALB/c mice according to guideline 425 by OECD: “Up-and-Down” method. AE: aqueous extract; FF: flavonoid fraction; i.p.: intraperitoneal; LD50: median lethal dose.
