# Supplementary material for: Biological and toxicological evaluation of Rhus trilobata Nutt. (Anacardiaceae) used traditionally in mexico against cancer
Source: BMC Complement Altern Med. 2019 Jul 1;19:153. doi: 10.1186/s12906-019-2566-9 (PMC6604276; doi:10.1186/s12906-019-2566-9)
Supplement: Supplementary file 2 — Figure S2. MS/MS analysis of the most abundant compounds in RHTR. (PPTX 1780 kb) [file 12906_2019_2566_MOESM2_ESM.pptx]

## Slide 1
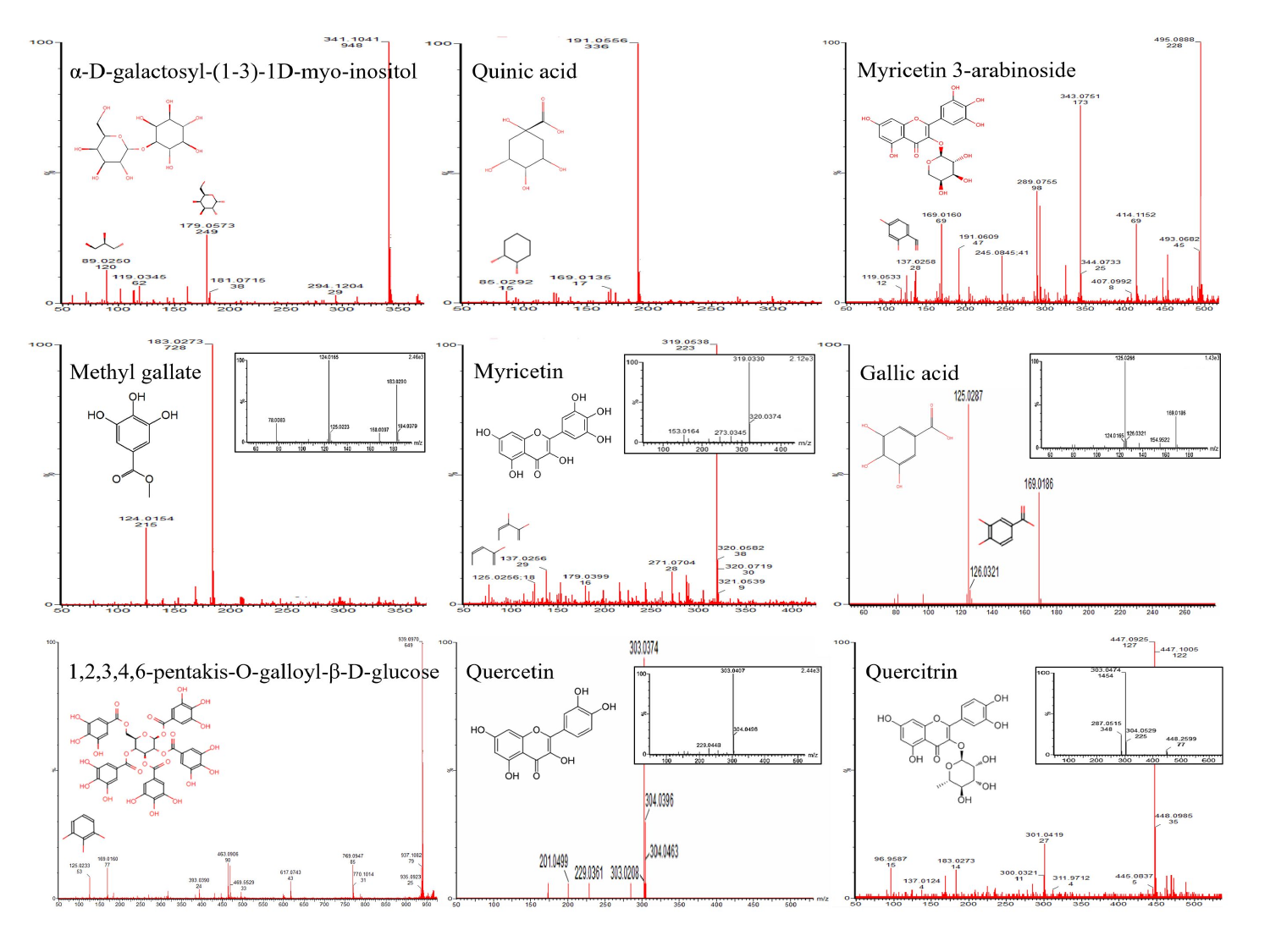

## Slide 2
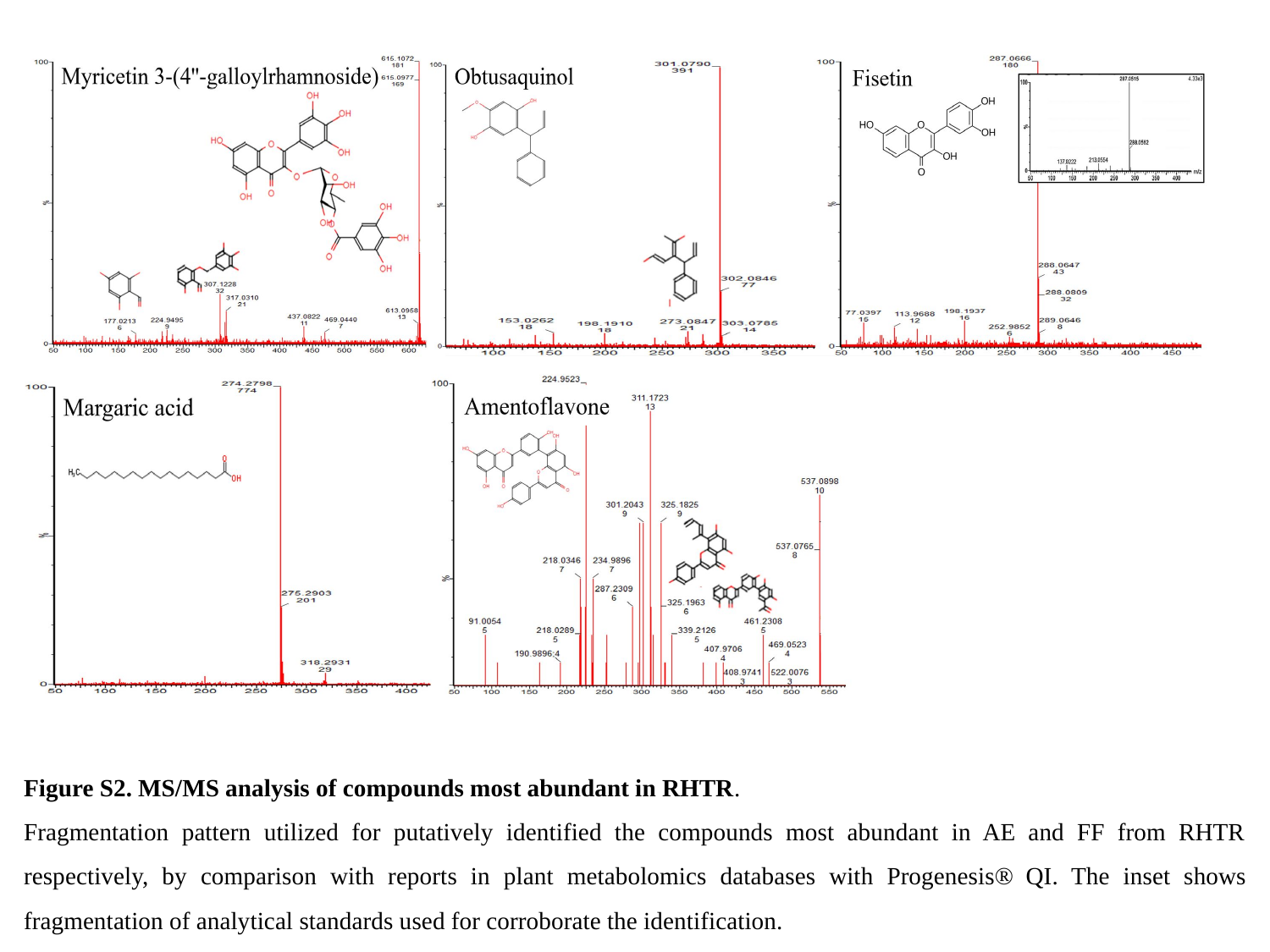

Figure S2. MS/MS analysis of compounds most abundant in RHTR.
Fragmentation pattern utilized for putatively identified the compounds most abundant in AE and FF from RHTR respectively, by comparison with reports in plant metabolomics databases with Progenesis® QI. The inset shows fragmentation of analytical standards used for corroborate the identification.
